# Tracking Youth Self-Inflicted Injury Hospitalizations to Target High-Risk Communities, Leverage Resources, and Unify Stakeholder Efforts: Illinois Department of Public Health

**DOI:** 10.5888/pcd11.140276

**Published:** 2014-11-13

**Authors:** Benjamin S. Arbise, Nancy L. Amerson

**Affiliations:** Author Affiliation: Nancy L. Amerson, Illinois Department of Public Health, Office of Health Promotion, Division of Chronic Disease Prevention and Control, Springfield, Illinois.

**Figure:**
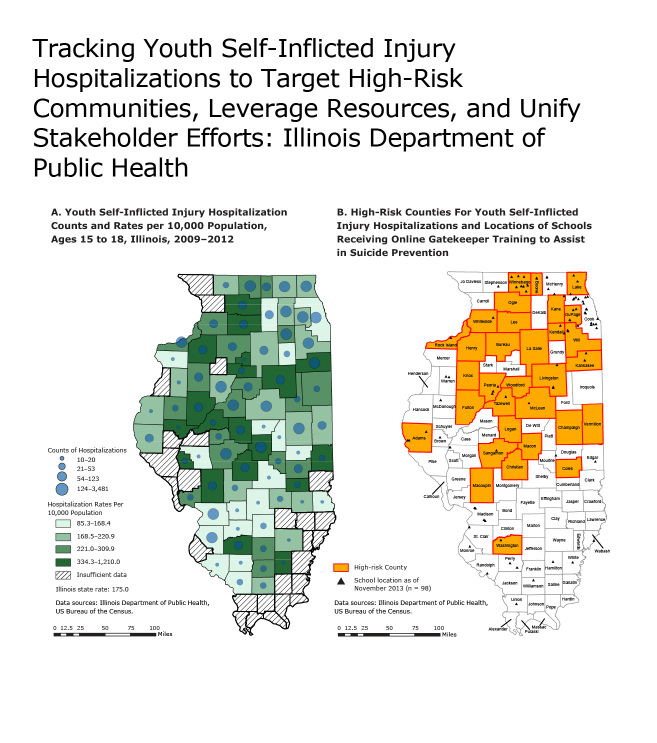
Working to expand youth suicide prevention activities, the Illinois Department of Public Health’s Youth Suicide Prevention Project developed these maps to 1) illustrate the burden of self-inflicted injury among youth aged 15 to 18, 2) display the locations of schools receiving Gatekeeper trainings, and 3) to identify high-risk counties for self-inflicted injury and future suicides. Using inpatient and outpatient hospitalization counts and rates (Map A), counties were identified as high-risk for youth self-inflicted injury (Map B). Used together, these maps will allow stakeholders to expand the reach of Gatekeeper trainings by increasing high school recruitment in high-risk counties with no current participation. Notes: A high-risk county has a hospitalization rate above the state average and a hospitalization count in the top 2 quartiles. Cook County is considered a high-risk county because its hospitalization count is the highest in the state (n = 3,481).

## Background

For every adolescent suicide completion in Illinois, there are an estimated 100 adolescent suicide attempts measured by self-inflicted injury (1). The Illinois Department of Public Health’s (IDPH’s) Youth Suicide Prevention Project (YSPP) sought to describe the burden of self-inflicted injury among youth at the county level by analyzing the number of hospitalizations (inpatient and outpatient) and the rates of hospitalizations among various youth populations (2,3). These data will be used in conjunction with program data to drive program decisions, identify high-risk counties, and market the interactive online Gatekeeper training program provided to IDPH by Kognito Solutions. Gatekeeper training sessions are research-proven simulations to prepare middle school and high school administrators, teachers, and staff to recognize psychological distress in students and link them with support systems. In Illinois, these training sessions started in 2012 and are funded through a grant from the Substance Abuse and Mental Health Services Administration. In addition to IDPH, which administers the grant, project stakeholders are the University of Illinois Center for Prevention Research, the Illinois Suicide Alliance, Kognito Solutions (provider of Gatekeeper training services), the Illinois State Board of Education, and various school districts in Illinois.

## Methods

Illinois hospital discharge data on self-inflicted injury from 2009 through 2012 among youth aged 15 to 18 were aggregated to the county level. Hospitalization data are tagged with supplemental codes (E codes) to capture information on external cause of injury and intent. The E codes used in this analysis were codes E950 through E959. Hospitalization rates for self-inflicted injury were calculated for each county using the number of youth aged 15 to 18 (according to the US Bureau of the Census [3]) for the denominators. ArcGIS 10.2.2 for desktop (ESRI), a geographic information system (GIS) software package, was used to map the counties in Illinois at highest risk for youth self-inflicted injury by using both hospitalization rates and counts. High-risk counties were defined as any county that has both high hospitalization rates and high hospitalization counts. High hospitalization rates due to self-inflicted injury were defined as rates above the state average (175.0 per 10,000 population). High hospitalization counts due to self-inflicted injury were defined as counts in the top 2 quartiles (54 to 3,481 self-inflicted injuries). Additionally, a GIS address geocoder was used to plot the addresses of Illinois schools participating in the online Gatekeeper training program. To increase reliability of the county estimates, counties with fewer than 10 cases of self-inflicted injury were removed, and their counts and rates were suppressed in the map.

## Main Findings

The mapping analysis identified 32 high-risk counties for youth aged 15 to 18. Most of the high-risk counties are in the central and northern regions of the state. The southern region of the state has only 1 high-risk county. Among the 32 high-risk counties, 17 counties had high schools that had participated in the Gatekeeper training program. As of November 2013, 98 high schools in Illinois had participated in the Gatekeeper training program.

## Action

YSPP plans to disseminate these maps to stakeholders to ensure that high-risk counties have access to data on the burden of suicide and to mobilize stakeholders to recruit school districts and high schools in high-risk communities for future Gatekeeper training. The coordination of stakeholders’ efforts and the expansion of the Gatekeeper training to high-risk counties will help increase the impact of suicide prevention in areas with the greatest need.
